# Supplementary material for: Different Traits, Different Evolutionary Pathways: Insights from Salamandrina (Amphibia, Caudata)
Source: Animals (Basel). 2022 Nov 28;12(23):3326. doi: 10.3390/ani12233326 (PMC9739870; doi:10.3390/ani12233326)
Supplement: Supplementary file 1 [file animals-12-03326-s001.zip › animals-2031885-supplementary.pdf]

Different traits, different evolutionary pathways: insights from *Salamandrina* (Amphibia, Caudata)

Claudio Angelini*, Francesca Antonucci, Jacopo Aguzzi, Corrado Costa
*corresponding author: oppela@gmail.com

Supplementary materials

Table S1. Data summary (mm) for the measured body features (see Fig. 1) of *Salamandrina*. Codes: populations as in the main text; species: Sp: *Salamandrina perspicillata*, St: *Salamandrina terdigitata*; habitat: BR: brooks, SW: lentic waters. For each group it is reported: sample size (within round brackets), and mean±SE; range is only reported for the populations (square brackets).

trait	Population	Species	Habitat
HL	AC (28) 10.8±0.1 [9.7 – 11.8]	Sp (72) 10.7±0.1	BR (108) 9.8±0.1
	CN (18) 11.3±0.1 [10.3 – 12.4]	St (82) 9.7±0.1	SW (46) 11±0.1
	SA (26) 10.3±0.1 [9.6 – 12]		
	TC (41) 9.7±0.1 [8.7 – 10.7]		
	TR (41) 9.6±0.1 [7.9 – 10.7]		
HW	AC (28) 7.48±0.06 [7 – 8.4]	Sp (72) 7.5±0.1	BR (108) 6.8±0.07
	CN (18) 8.29±0.06 [7.7 – 8.7]	St (82) 6.7±0.1	SW (46) 7.8±0.03
	SA (26) 7.07±0.06 [6.5 – 7.9]		
	TC (41) 6.61±0.4 [6.1 – 7]		
	TR (41) 6.73±0.05 [5.8 – 7.4]		
No	AC (28) 1.91±0.05 [1.1 – 2.6]	Sp (72) 1.96±0.03	BR (108) 1.86±0.01
	CN (18) 2.13±0.05 [1.7 – 2.4]	St (82) 1.84±0.02	SW (46) 1.99±0.03
	SA (26) 1.89±0.03 [1.6 – 2.2]		
	TC (41) 1.81±0.03 [1.2 – 2.1]		
	TR (41) 1.88±0.02 [1.5 – 2.1]		
AG	AC (28) 22.51±0.31 [19.65 - 25.25]	Sp (71) 22.58±0.28	BR (100) 19.85±0.22
	CN (18) 25.35±0.29 [22.6 - 26.95]	St (75) 19.58±0.27	SW (46) 23.62±0.3
	SA (25) 20.66±0.33 [18.25 – 25.2]		
	TC (43) 18.56±0.35 [15 – 27.05]		
	TR (32) 20.95±0.27 [18.2 - 23.5]		
CL	AC (28) 3.36±0.09 [2 - 4.3]	Sp (71) 3.28±0.06	BR (100) 3.02±0.04
	CN (18) 3.55±0.14 [2.35 - 4.55]	St (75) 3.03±0.04	SW (46) 3.43±0.08
	SA (25) 3±0.09 [1.8 – 3.7]		
	TC (43) 3.09±0.04 [2.35 - 3.55]		
	TR (32) 2.96±0.07 [2.25 - 3.8]		
tL	AC (28) 57.82±0.7 [52.75 – 65.8]	Sp (70) 56.970.67	BR (100) 48.65±0.52
	CN (18) 62.49±0.94 [54.55 – 71.95]	St (75) 47.51±0.58	SW (45) 59.63±0.66
	SA (25) 52.07±0.83 [46.5 – 60.65]		
	TC (43) 44.65±0.56 [35.35 – 53.75]		

	TR (32) 51.35±0.69 [40.9 – 60.05]		
tW	AC (28) 3.24±0.04 [2.9 - 3.7]	Sp (70) 3.28±0.05	BR (100) 3.43±0.06
	CN (17) 3.75±0.11 [3.25 - 5.15]	St (75) 2.87±0.02	SW (45) 2.9±0.02
	SA (25) 3±0.05 [2.6 – 3.55]		
	TC (43) 2.89±0.03 [2.45 – 3.55]		
	TR (32) 2.84±0.03 [2.5 - 3.25]		
tH	AC (28) 3.23±0.04 [2.8 - 3.65]	Sp (70) 3.31±0.05	BR (100) 3.12±0.03
	CN (17) 3.72±0.12 [3 - 4.6]	St (75) 3.12±0.03	SW (45) 3.41±0.06
	SA (25) 3.13±0.07 [2.7 – 4.3]		
	TC (43) 3.07±0.04 [2.6 – 3.9]		
	TR (32) 3.18±0.04 [2.6 - 3.6]		
RU	AC (28) 4.98±0.05 [4.5 - 5.6]	Sp (68) 4.97±0.04	BR (99) 4.5±0.03
	CN (15) 5.31±0.07 [4.85 - 5.8]	St (74) 4.41±0.03	SW (43) 5.09±0.05
	SA (25) 4.77±0.07 [4.2 – 5.75]		
	TC (43) 4.33±0.03 [3.9 – 4.85]		
	TR (31) 4.52±0.04 [4.15 - 5.1]		
TF	AC (28) 4.68±0.05 [4.15 - 5.1]	Sp (68) 4.69±0.05	BR (99) 4.2±0.03
	CN (15) 5.18±0.06 [4.7 - 5.65]	St (74) 4.12±0.03	SW (43) 4.85±0.05
	SA (25) 4.41±0.06 [3.95 – 5.1]		
	TC (43) 4.04±0.04 [3.5 – 4.7]		
	TR (31) 4.23±0.04 [3.75 - 4.65]		
SVL	AC (28) 39.19±0.37 [35.4 - 43]	Sp (71) 39.56±0.39	BR (100) 35.49±0.28
	CN (18) 43.66±0.37 [40 - 47.1]	St (75) 34.97±0.31	SW (46) 40.94±0.42
	SA (25) 37.03±0.49 [33.9 – 42.45]		
	TC (43) 33.62±0.85 [29.5 – 37.95]		
	TR (32) 36.79±0.35 [33.05 - 40.05]		
TL	AC (27) 100.42±1.01 [91.2 - 112.2]	Sp (70) 99.8±1.1	BR (100) 87.16±0.77
	CN (18) 109.7±1.21 [97.9 - 121.95]	St (75) 85.51±0.86	SW (45) 104.13±1.03
	SA (25) 92.1±1.28 [84.3 – 105.15]		
	TC (43) 81.36±0.85 [68.8 – 94.55]		
	TR (32) 91.09±1.02 [76.5 - 101.95]		

Table S2 Candidate models analysing the dependence of snout-vent length of salamanders basing on their species (*Salamandrina perspicillata* or *S. terdigitata*), the typology of the breeding site (brook or still water) and their population (five populations). Species and site typology have been used as fixed factors, population as random factor. AICc is the Akaike's information criterion for the small sample size, w is the model weight.

model	AICc	w
site typology:population	662.19	0.68
species:population	664.56	0.21
population	665.87	0.11
site typology	720.34	0
species	740.38	0

Table S3 Candidate models analysing the dependence of total length of salamanders basing on their species (*Salamandrina perspicillata* or *S. terdigitata*), the typology of the breeding site (brook or still water) and their population (five populations). Species and site typology have been used as fixed factors, population as random factor. AICc is the Akaike's information criterion for the small sample size, w is the model weight.

model	AICc	w
site typology:population	937.17	0.75
species:population	940.03	0.18
population	941.98	0.07
site typology	998.46	0
species	1023.69	0

Supplementary material S4: example of further analysis

We analysed the head colour pattern by using clustering methods. Since this method does not reduce dimensionality and does not identify the main features underlining differences, we applied a multivariate classification analysis, the Partial Least Square Discriminant Analysis (PLSDA) to the information on position and colour (black or white) of pixels of the head colour pattern. PLSDA is a PLS regression where the response variable is categorical, expressing the class membership of the statistical units (Sjöström et al., 1986; Sabatier et al., 2003; Costa et al., 2013; Infantino et al., 2015). The objective of PLSDA is to find a model, developed from a training set of observations of known class membership that separates classes of objects basing on their X-variables. PLSDA was used as a supervised modelling class method (Forina et al., 2008a,b), using SIMPLS algorithm (de Jong, 1993) to classify samples. The dataset was divided into a calibration set composed by 75% of individuals and an internal validation set represented by the remaining 25%. This partition was done by optimally choosing the Euclidean distances based on the algorithm of Kennard and Stone (1969). That algorithm selects objects without the a priori knowledge of a regression model. The percentages of correct classification were calculated for calibration and validation phases, and then used for model selection. We based PLSDA model selection on RMSEC, RMSECV and the discrimination efficiencies and robustness of used parameters, namely the sensitivity and the specificity. The sensitivity is the percentage of the individuals of a category accepted by the class model. The specificity is the percentage of the individuals of the categories different from the modelled one, rejected by the class model. This analysis was performed using Matlab (rel. 7.1, PLSToolbox Eigenvector rel. 4.0) on the single pixel colour variables (X-block) on the superimposed configuration of each individual based on the head shape morphometric analysis.

Table S4 shows the characteristics and the main results of the selected PLSDA models performed on the single pixel colour variables (X-block) on the superimposed configuration of each individual for the grouping variables -populations, species, and habitats- constructed with 10, 5, and 5 latent vectors respectively. In all calibrations, the three models based on population, species and habitat, get the highest specificity and sensitivity and the lowest classification error, and correctly classified all the individuals. The independent test for the population grouping had a 62.2% percentage of correct classification (probability of random assignment: 20%). The model constructed to discriminate individuals of S. *perspicillata* and S. *terdigitata* returned a percentage of correct classification of 73.7% (probability of random assignment: 50%); the model built to discriminate between individuals from lentic water and brook habitats correctly classified 71.1% of them (probability of random assignment: 50%).

Table S4

	Populations	Species	Habitats
individuals for 75% modelling and validation	119	118	118
individuals for 25% independent test	37	38	38
classes	5	2	2
random probability (%)	20	50	50
number of latent vectors	10	5	5
% cumulated variance X-block	34.72	22.87	25.94
% cumulated variance Y-block	66.68	40.26	41.36
pre-processing X-block	Mean center	Mean center	Mean center
pre-processing Y-block	none	none	none
mean specificity (%)	100	100	100
mean sensitivity (%)	100	100	100

mean classification error (calibration)	0	0	0
% correctly classified model	100	100	100
misclassified individuals (model 75%)	0	0	0
% correctly classified independent test	62.16	73.68	71.05
misclassified individuals (test 25%)	14	10 (8 for *S. terdigitata*)	11 (9 for pools)

As an example, figure S4b shows the loadings of each pixels for each latent variable from the PLSDA performed on the head colour pattern for the grouping variable species. White intensity is related to a higher contribution to the classification.

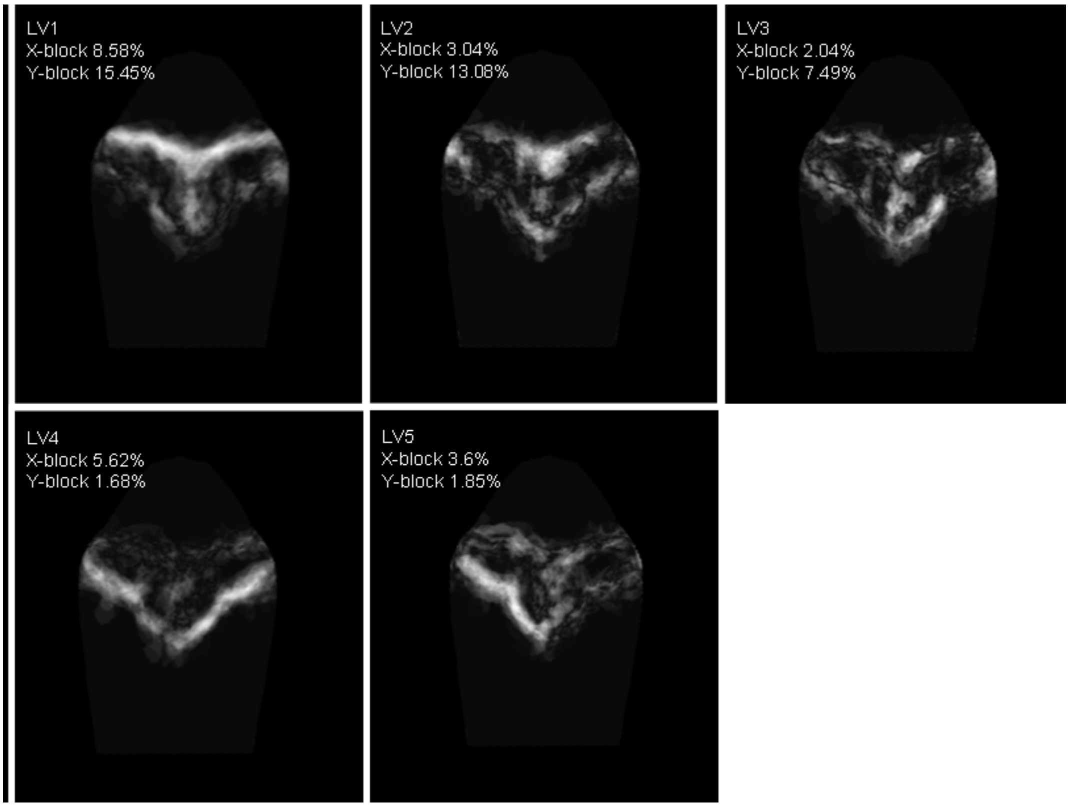

Figure S1

References

Costa, C.; Antonucci, F.; Boglione, C.; Menesatti, P.; Vandeputte, M.; Chatain, B. Automated sorting for size, sex and skeletal anomalies of cultured seabass using external shape analysis. *Aquacult. Eng.* **2013**, *52*, 58-64.

de Jong, S. SIMPLS: an alternative approach to partial least squares regression. *Chemometr. Intell. Lab.* **1993**, *18*, 251-263.

Forina, M.; Oliveri, P.; Casale, M.; Lanteri, S. Multivariate range modeling, a new technique for multivariate class modeling: the uncertainty of the estimates of sensitivity and specificity. *Anal. Chim. Acta* **2008**, 622, 85-93.

Forina, M.; Oliveri, P.; Lanteri, S.; Casale, M. Class-modeling techniques, classic and new, for old and new problems. *Chemometr. Intell. Lab.* **2008**, *93*, 132-148.

Infantino, A.; Aureli, G.; Costa, C.; Taiti, C.; Antonucci, F.; Menesatti, P.; Pallottino, F.; De Felice, S.; D'Egidio, M.G.; Mancuso, S. Potential application of PTR-TOFMS for the detection of deoxynivalenol (DON) in durum wheat. *Food Control* **2015**, *57*, 96-104.

Kennard, R.W.; Stone, L.A. Computer aided design of experiments. *Technometrics* **1969**, *11*, 137–148.

Sabatier, R.; Vivein, M.; Amenta, P. Two approaches for discriminant partial least square. In: *Between data science and applied data analysis*, Schader, M., Gaul, W., Vichi, M. Eds; Springer-Verlag, Berlin, 2003, pp. 100-108.

Sjöström, M.; Wold, S.; Söderström, B. PLS discrimination plots. In: *Pattern recognition in practice II*, Gelsema, E.S., Kanals, L.N. Eds.; Elsevier, Amsterdam, 1986, pp. 461-470.
